# 

**Published:** 1882-04

**Authors:** 


					﻿PAMPHLETS RECEIVED :
The Vienna Obstetrical School, and 300 consecutive Obstetric
Cases. By M. M. Walker, M. D.
Epilepsy; its Relations to Crime. By J. Martine Kershaw, M.D.
A Directory of Homoeopathic Physicians in Pennsylvania. By
L. J. Knerr, M. D.
Head Symptoms, Before, During and after Menstruation. By
Henry Minton, M. D. (See Periscope.)
NOTICE.
All articles for publication, books for review, business communications} etc., '
must be sent to Editor, 210!) Chestnut Street, Philadelphia.
CONTRIBUTORS:
Dirs. H. C. Allen, T. F. Allen, Edward Bayard, J. B. Bell, E. W. Berridge,
Geo. JET. Clark, A. C. Cowperthwaite, Ad. Fellger, Wm. J. Guernsey,
W. A- Hawley, John Hall, W. M. James, W. H. Jenny, Ad. Lippe,
C. Lippe, Malcolm MacFarlan, Thos. Moore, C. F. Nichols,
. . ‘ C. Pearson, T. F. Pomeroy, Leila A. RenDell, C, Carle-,
■ ton Smith, P. P. Walls, Wm. P. Wesselhoeft,
David Wilson, (London), T. P. Wilson,
> and marly others.
Authors of accepted papers are furnished with copies of the
number in which their articles appear; application for such>copies ,
; t<r be made when sending papers. Any irregularity in the receipt
of this Journal should be promptly reported to Editor.
				

